# The CALHM1 blocker CGP37157 increases seizure severity during status epilepticus in adult mice

**DOI:** 10.1007/s11302-025-10103-9

**Published:** 2025-07-02

**Authors:** Meghma Mitra, Amaya Sanz Rodriguez, Norman Delanty, Alan Beausang, Francesca M. Brett, Michael A. Farrell, Jane Cryan, Donncha F. O’Brien, David Henshall, Maria F. Cano-Abad, Tobias Engel

**Affiliations:** 1 Department of Physiology & Medical Physics, RCSI University of Medicine & Health Sciences, Dublin, D02 YN77 Ireland; 2 FutureNeuro Research Ireland Centre, RCSI University of Medicine and Health Sciences, Dublin, D02 YN77 Ireland; 3 Department of Neurology, Beaumont Hospital, Dublin, Ireland; 4 Department of Neuropathology, Beaumont Hospital, Dublin, Ireland; 5 Department of Neurosurgery, Beaumont Hospital, Dublin, Ireland; 6 Departamento de Farmacología, Facultad de Medicina, Universidad Autónoma de Madrid, Madrid, Spain; 7 Instituto de Investigación Sanitaria, Hospital Universitario de La Princesa, Universidad Autónoma de Madrid, Madrid, Spain

**Keywords:** CALHM1, Status epilepticus, Epilepsy, Intra-amygdala kainate model, Resected tissue from TLE patients

## Abstract

Epilepsy is one of the most common chronic brain diseases affecting up to 70 million people worldwide. Major challenges of epilepsy treatment include the high pharmacoresistance in patients and the lack of disease-modification. Extracellular adenosine 3’triphosphate (ATP), a key neurotransmitter in the activation of the purinergic signalling system, is increasingly recognized to contribute to pathological brain hyperexcitability in epilepsy. Consequently, targeting ATP-release mechanisms may constitute a new therapeutic strategy for seizure control and epilepsy. The calcium channel, Calcium Homeostasis Modulator 1 (CALHM1), a voltage-gated, non-selective ion channel that permits the passage of various cations and small molecules, is expressed in neurons and plays an essential role during neuronal excitability and neurotransmission. In addition to ions, CALHM1 also allows the passage of ATP into the extracellular space, activating thereby purinergic receptors. Here, we tested if the pharmacological blocking of CALHM1 via CGP37157 (7-chloro-5-(2-chlorophenyl)-3,5-dihydro-4,1-benzothiazepin-2-(1H)-one) alters the severity of intra-amygdala kainic acid-induced status epilepticus. Our results show that CGP37157 increased the severity of seizures during status epilepticus. In addition, CALHM1 protein levels are down-regulated in the hippocampus in epileptic mice and Temporal Lobe Epilepsy (TLE) patients. In summary, our results identify CALHM1 as a new contributor to seizures and suggest targeting of CALHM1 as new treatment strategy for epilepsy.

**Supplementary Information:**

The online version contains supplementary material available at 10.1007/s11302-025-10103-9.

## Introduction

Epilepsy, characterized by recurrent spontaneous seizures, is one of the most common chronic brain diseases affecting up to 70 million people worldwide [1]. Epilepsy can be caused by genetic abnormalities or acquired following an insult to the brain (*e.g.*, traumatic brain injury (TBI), status epilepticus (SE)). Anti-seizure medications (ASMs) remain the mainstay of epilepsy treatment with over 30 ASMs in clinical use [1]. To date, 30% of patients remain, however, refractory to any pharmacological interventions and, even if effective in suppressing seizures, current ASMs do not alter significantly the course of the disease and can cause serious adverse side effects [2].

Purinergic signalling via extracellularly released ATP (eATP) is increasingly recognized to contribute to pathological brain hyperexcitability [3]. eATP concentrations, usually found at low tissue concentrations (nanomolar range), increase sharply upon pathological insults (*e.g*., a seizure [4, 5]) in the extracellular space (millimolar range) activating specific membrane receptors including the metabotropic P2Y and ionotropic P2X receptors [6]. Data generated over the past decade has demonstrated a clear role of these receptors not only in the generation of seizures but also during the development of epilepsy [3], providing the rationale for targeting ATP release mechanisms.

eATP is tightly regulated via different ATP release and degradation mechanisms [7, 8] including the action of ATP-degrading ectonucleotidases and exocytotic and non-exocytotic mechanisms such as the Cl^−^-dependent vesicular nucleotide transporter (VNUT), voltage-dependent anion channels, ATP-binding cassette transporters, and hemichannels such as connexins and pannexins.

The calcium channel Calcium Homeostasis Modulator 1 (CALHM1) has several structural and functional similarities to connexins, pannexins, and innexins [9]. CALHM1 has been proposed to be the pore-forming subunit of an ion channel, which modulates synchronized neuronal excitability in cooperation with extracellular Ca^2+^ [10]. CALHM1, mainly expressed in neurons, has been found located in the plasma membrane, but also in the endoplasmic reticulum (ER), where it plays a role in alterations of Ca^2+^ homeostasis, ER-stress, and cell toxicity [11]. CALHM1 channels are permeable to cations including Ca^2^⁺, Na⁺, and K⁺, and to a lesser extent Cl⁻. CALHM1 has also been shown to form a pore capable of permeating ATP, possibly contributing to the eATP pool and the activation of ATP-gated P2 receptors [10, 12, 13]. These dual functions suggest roles in both ionic conductance and purinergic signalling [14].

To date, CALHM1 function has been linked to several pathologies of the CNS including Alzheimer’s disease [15] and cerebral ischemia [16]. While previous research has suggested CALHM1 polymorphisms to be associated with TLE [17], whether CALHM1 function is involved in seizures has not been investigated to date.

## Materials and methods

### Animals

All animal studies adhered to the principles of the European Communities Council Directive (2010/63/EU), and relevant national licenses were approved by the Research Ethics Committee of the Royal College of Surgeons in Ireland (RCSI) (REC 1322) and the Irish Products Regulatory Authority (AE19127/P038). 8–12-week FVB/NJ wild-type male mice were sourced from the Biomedical Research Facility (BRF, RCSI, Dublin, Ireland). Mice were housed in groups of 2–5 per cage and kept in a controlled animal facility on a 12 h light/dark cycle at 22 ± 1ºC and humidity of 40–60%.

### Status epilepticus mouse model

Status epilepticus (SE) was induced as described previously [18]. Isoflurane anesthetized mice (5% induction, 1–2% maintenance) were placed in a stereotaxic frame and a midline scalp incision was performed to expose the skull. To minimize pain during and post-surgery, mice were treated with buprenorphine (0.05 mg/kg) and EMLA cream (Aspen Pharma, UK). A guide cannula for intra-amygdala kainic acid (IAKA) injection (coordinates from Bregma; AP = −0.94 mm, L = −2.85 mm) and three cortical electrodes, one on top of each hippocampus and the reference electrode on top of the frontal cortex, were fixed in place with dental cement. Approximately 1 h post-EEG electrode and cannula implantation, SE was induced in awake, hand-restrained mice, by a microinjection of 0.2 µg KA in 0.2 µl phosphate-buffered saline (PBS) (Sigma-Aldrich, Dublin, Ireland) into the right basolateral amygdala. Vehicle-injected control animals received 0.2 µl of PBS (pH = 7.4) solution. The anticonvulsant lorazepam (6 mg/kg) (Wyetch, Taplow, UK) was delivered intraperitoneal (i.p.) 40 min following IAKA or vehicle to curtail seizures and reduce morbidity and mortality.

### Drug administration

CGP37157 [15] was delivered by an intracerebroventricular (i.c.v.) injection (2 µl) 10 min prior to IAKA injection via a previously implanted cannula fixed with dental cement (coordinates from Bregma; AP = −0.4 mm, L = −1 mm, depth 2 mm). Animals were divided into three treatment groups: (i) control, injected with vehicle (PBS), (ii) mice receiving 1 µM of CGP37157, and (iii) mice receiving 10 µM of CGP37157.

### Seizure severity analysis

An electroencephalogram (EEG) was recorded using an Xltek recording system (Optima Medical Ltd, Guildford, UK) [18]. To analyze EEG frequency and amplitude signals, EEG data were uploaded into Labchart7 software (AD Instruments Ltd, Oxford, UK). Seizure onset was analyzed offline. Seizure onset was defined as first seizure burst detectable on the EEG consisting of high amplitude (> twice baseline) high frequency polyspiking of a minimum of 5 s in duration. The duration of high-frequency (> 5 Hz) and high-amplitude (HFHA) (> 2 times baseline) polyspike discharges of ≥ 5 s duration was also counted manually by a reviewer blinded to treatment.

### Human brain tissue

All subjects gave their informed consent for inclusion before they participated in the study. The study was conducted in accordance with the Declaration of Helsinki, and the protocol was approved by the Ethics Committee of Beaumont Hospital, Dublin (05/18). Briefly, patients (*N = *10) were referred for surgical resection of the temporal lobe for the treatment of intractable TLE. After temporal lobe resection, cortex and hippocampi were obtained from the same patient and frozen in liquid nitrogen and stored at −80 °C until use. A pathologist assessed hippocampal tissue and confirmed the absence of significant neuronal loss. Control (autopsy) temporal hippocampi (*N = *5) and cortex (*N = *5) were obtained from individuals from the Brain and Tissue Bank for Developmental Disorders at the University of Maryland, Baltimore, MD, U.S.A. Brain sample and donor metadata are available in Supplementary Table 1.

### Western blotting

Western blot analysis was performed as described previously [18]. Lysis buffer (100 mM NaCl, 50 mM NaF, 1% Tx-100, 5 mM EDTA pH 8.0, 20 mM HEPES pH 7.4) containing a cocktail of phosphatase and protease inhibitors was used to homogenize hippocampal brain tissue and to extract proteins. Following electrophoresis, proteins were transferred to a nitrocellulose membrane (GE Health Care, Illinois, USA) and immunoblotted with CALHM1 antibody (Proteintech Europe) (1:1000, prepared in 5% milk- tris-buffered saline-tween (TBST)). Membranes were incubated with horseradish peroxidase-conjugated goat anti-rabbit (1:5000, prepared in 5% milk-TBST, Sigma-Aldrich, Dublin, Ireland). Protein bands were visualized using Fujifilm LAS-4000 system with chemiluminescence (Immombilon Western HRP substrate, Merck Millipore, Massachusetts, USA) followed by analysis using ImageJ. Protein quantity was normalized to the loading control GAPDH (1:5000 prepared in 5% milk-TBST; anti-mouse; Sigma-Aldrich) or β-Actin (1:5000 prepared in 5% milk-TBST; anti-mouse; Sigma-Aldrich).

### Statistical analysis

Statistical analysis of data was carried out using GraphPad Prism 8 and STATVIEW software (SAS Institute, Cary, NC, U.S.A). Analysis of variance (ANOVA) with post hoc Fisher’s protected least significant difference was used to analyze group data of three or more. For two-group comparisons, Student’s t-test was used to determine statistical differences between groups. Normality and lognormality test were used to verify the normal distribution between groups. Outliers were identified according to the Grubbs’ test. Significance was accepted at *P < 0.05.

## Results

### Reduced CALHM1 protein levels in the hippocampus in epilepsy

**Fig. 1:**
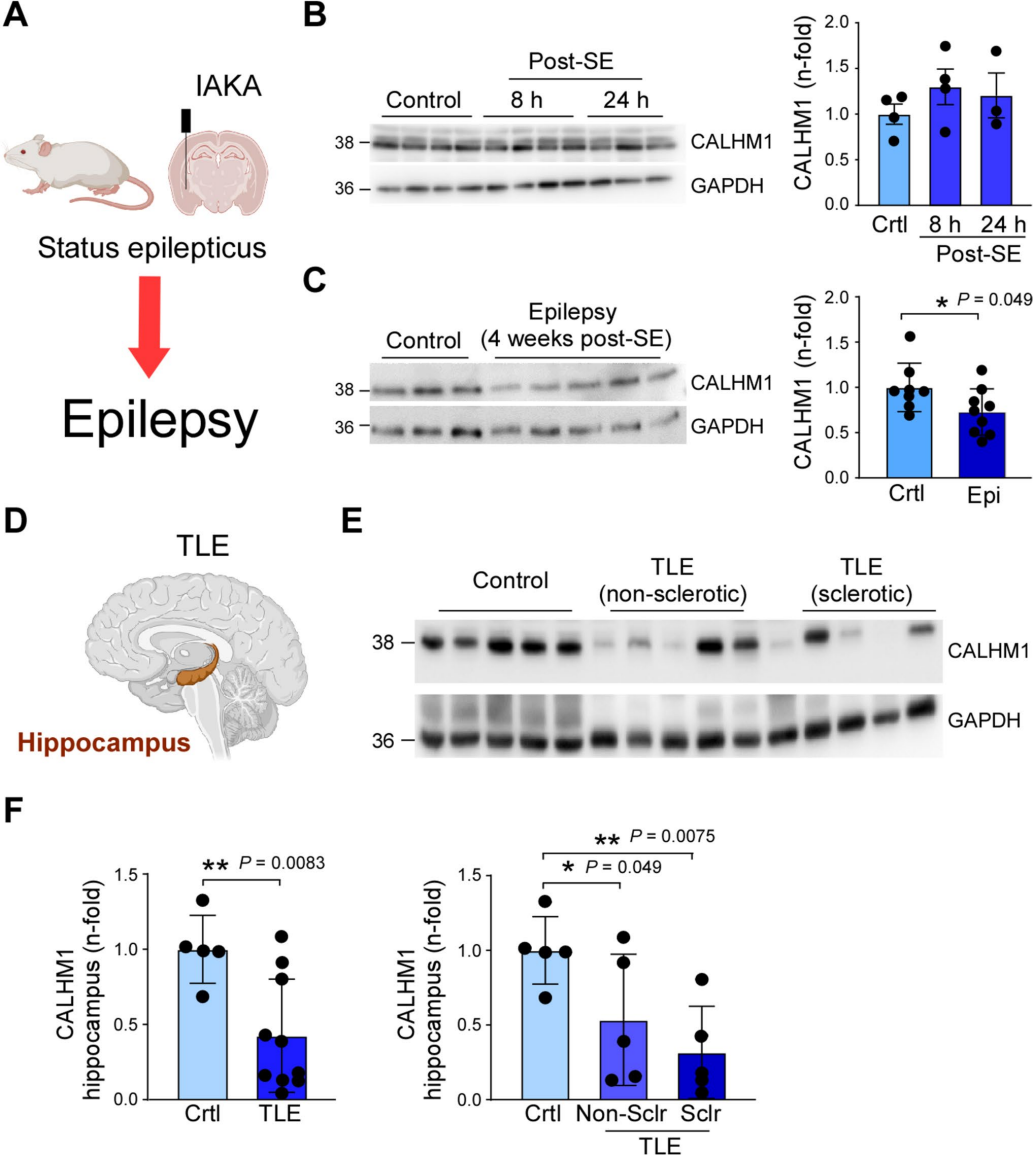
CALHM1 protein levels in the hippocampus in epilepsy. **A** Mice subjected to IAKA develop epilepsy after a short latency period of 2–3 days. **B** Western blot and graph showing CALHM1 protein levels in the hippocampus 8 and 24 h post-SE (*N = *4 (control and 8 h post-SE) and 3 (24 h post-SE)). **C** Western blot and graph showing CALHM1 protein levels in the hippocampus of mice 4 weeks post-IAKA (*N = *8 (control) and 9 (epilepsy)). **D** Schematic showing hippocampus in the human brain. **E**, **F** Western blot and graphs showing CALHM1 protein levels in the hippocampus of healthy control and resected tissue from TLE patients with and without hippocampal sclerosis (*N = *5 per group). Data are shown as mean ± SD. *P < 0.05; **P < 0.01

To test whether CALHM1 protein levels are altered following SE and in epilepsy, we analyzed ipsilateral hippocampal tissue from mice subjected to IAKA-induced SE shortly following SE and once epilepsy was established. In the IAKA mouse model, mice develop epilepsy after a short latent period of 2–5 days (Fig. 1A) [19]. While no change in CALHM1 protein levels was observed 8 h and 24 h post-SE (Fig. 1B), CALHM1 protein levels were reduced 4 weeks post-IAKA (Fig. 1C), time-point when epilepsy is usually well-established in the IAKA model [19–21]. We next analyzed CALHM1 protein levels in resected brain tissue from TLE patients (Fig. 1D). Similar to our data from the IAKA mouse model, CALHM1 protein levels were reduced in the hippocampus of TLE patients with and without hippocampal sclerosis (Fig. 1E, F). No changes in CALHM1 protein levels were observed in the cortex from the same patients (Supplementary Fig. 1A). No correlation between post-mortem interval and CALHM1 protein levels in control was observed (Supplementary Fig. 1B). Moreover, a simulated post-mortem experiment in mice showed no significant changes in hippocampal CALHM1 protein levels 8 h post-mortem when compared to control (Supplementary Fig. 1C), suggesting post-mortem time interval not to impact protein levels.

#### Increased seizure severity during SE via treatment with the CALHM1 blocker CGP37157

**Fig. 2:**
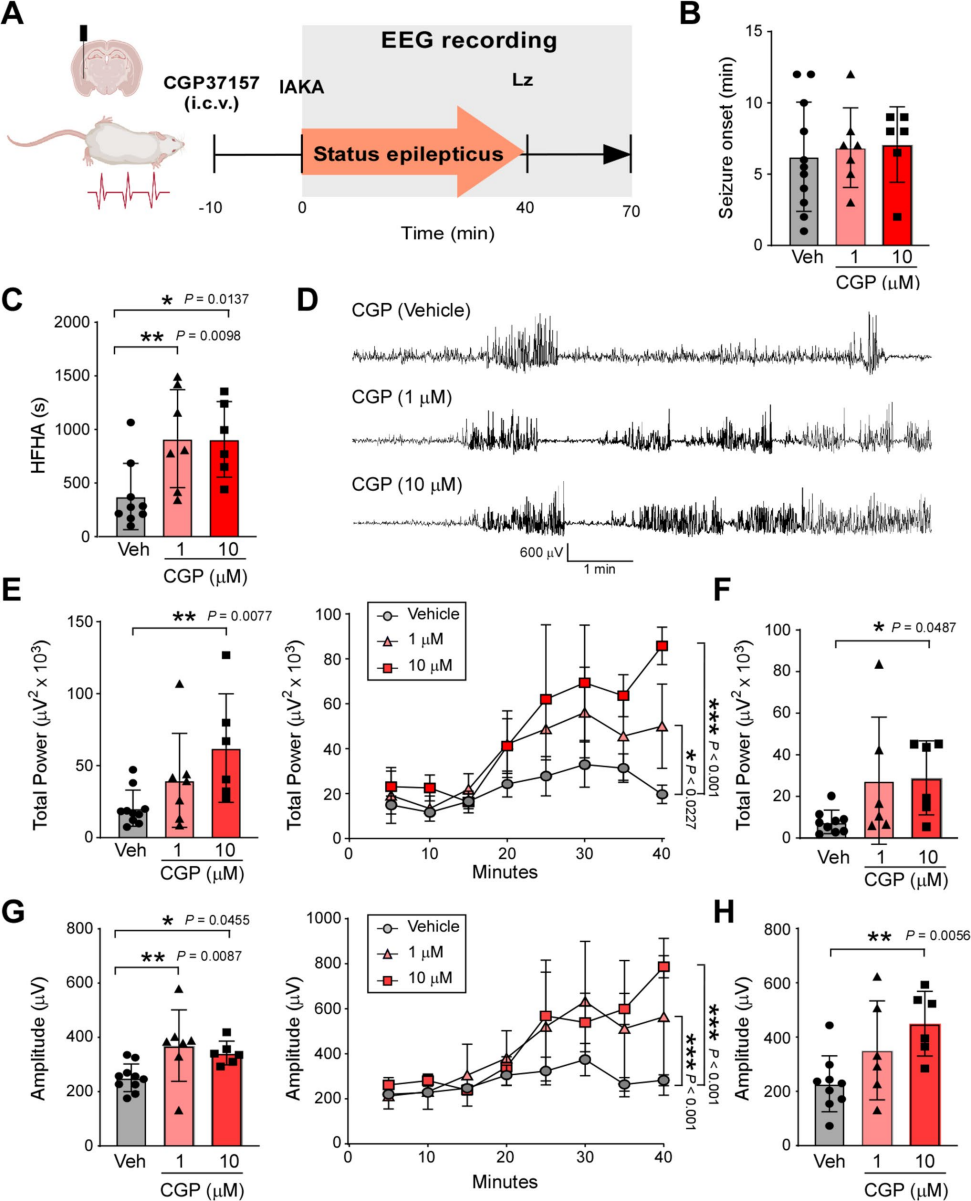
Effects on seizure severity during SE of the CALHM1 blocker CGP37157. **A** Schematic showing experimental design. Mice were i.c.v. injected with the CALHM1 blocker CGP37157 10 min before IAKA. EEG was recorded for 70 min starting at the time of IAKA injections until 30 min post-lorazepam treatment. Graphs showing seizure onset (**B**) (*N = *11 (Vehicle), 7 (1 μM CGP37157) and 6 (10 μM CGP37157) and total time of HFHA spiking (**C**) on the EEG from IAKA until lorazepam treatment (*N = *9 (Vehicle), 7 (1 μM CGP37157) and 6 (10 μM CGP37157)). **D** Representative EEG in mice treated with Vehicle or CGP37157 (CGP). **E** Total power on the EEG during a 40 min recording period starting at the time of IAKA (left: EEG analysis of complete 40 min; right: EEG analysis of 5 min segments) (*N = *9 (Vehicle), 7 (1 μM CGP37157) and 6 (10 μM CGP37157)). **F** Total power on the EEG during a 30 min recording period starting at the time of lorazepam treatment (*N = *9 (Vehicle), 6 (1 μM CGP37157) and 6 (10 μM CGP37157)). **G** EEG amplitude during a 40 min recording period starting at the time of IAKA (left: EEG analysis of complete 40 min; right: c) (*N = *9 (Vehicle), 7 (1 μM CGP37157) and 6 (10 μM CGP37157)). **H** EEG amplitude during a 30 min recording period starting at the time of lorazepam treatment (*N = *9 (Vehicle), 6 (1 μM CGP37157) and 6 (10 μM CGP37157)). Data are shown as mean ± SD except for E and G right graph where it is shown as mean ± SEM. *P < 0.05

To test whether blocking CALHM1 impacts seizure severity, mice were treated with CGP37157, which has previously been shown to block CALHM1 [15], i.c.v. 10 min before the injection of IAKA at two different doses (1 µM and 10 µM). Cortical EEG was recorded for 70 min starting at the time of IAKA (Fig. 2A). While no difference was observed in seizure onset between vehicle-injected mice and mice treated with CGP37157 (Fig. 2B), mice treated with CGP37157 (1 µM and 10 µM) showed an increase in HFHA spiking from the time of IAKA until the administration of lorazepam (Fig. 2C, D). Of note, CGP37157 on its own seemed to induce no obvious changes on the EEG (Supplementary Fig. 2A). No differences between treatment groups could be observed regarding frequency (Supplementary Fig. 2B). Mice treated with CGP37157 also showed an increased total seizure power and amplitude from the time of IAKA administration until treatment with the anticonvulsant lorazepam. This increase persisted for an additional 30 min post-lorazepam (Fig. 2E-H).

## Discussion

Here, we show that treatment with the CALHM1 blocker CGP37157 increases seizure severity during SE, suggesting CALHM1 activation as innate defence mechanism to protect the brain against a pathological brain hyperexcitability. Further analysis of brain tissue from epileptic mice and TLE patients showed decreased CALHM1 protein levels in the hippocampus.

The purinergic signalling system comprises a multitude of different components each intrinsically linked to each other. Whereas increasing evidence has demonstrated that the purinergic signalling system contributes to seizures and epilepsy [3], the contribution of each component to pathological brain hyperexcitability remains to be established. While the most focus has been put on receptors responding to extracellular released purines (e.g., P2X7R activated via eATP [22]), data also show a role for ATP release mechanisms. Of note, the deletion of pannexin-1 provided potent anticonvulsive effects during kainic acid (KA)-induced SE in mice and resected brain tissue from epilepsy patients [23]. Our study adds CALHM1, previously shown to be involved in ATP release [12], as a new player contributing to seizures.

Our data shows a strong increase in seizure severity mediated via the CALHM1 blocker CGP37157. The mechanism of how CALHM1 inhibition leads to an increase in seizure activity remains to be determined. While with the data provided it is not possible to pin-point the mechanism of action of how CALHM1 contributes to seizures, the regulation of eATP levels represents a potential possibility [5, 24]. Of note, while generally thought to be proconvulsant, recent research has also suggested an anticonvulsant function of eATP via the activation of ATP-gated channels (*e.g*., P2X7Rs expressed in inhibitory interneurons [18]. Thus, CALHM1inhibition may lead to a reduction in the eATP pool and, as a consequence, to a reduction in P2X7R-mediated inhibition. One should, however, remember that the purinergic signalling system is a complex signalling system comprising a multitude of different channels with almost all having been described to have a role during seizures [3]. Importantly, eATP can also be broken down into different metabolites including adenosine, which is a known anticonvulsant [25]. Moreover, in addition to the regulation of eATP, CALHM1 also regulates the passage of several ions including cations and anions [12], thus CALHM1 activation may impact purinergic signalling and ion flux across the cell membrane. Potentially adding further to the complexity of CALHM1 signalling, while CALHM1 has previously been shown to be expressed on excitatory cortical and hippocampal pyramidal neurons [10], its expression has not been excluded in inhibitory interneurons. Moreover, its cell-type specific expression and function may change during seizures and epilepsy. Therefore, depending on the neuronal subtype involved (excitatory vs inhibitory neurons), CALHM1-depedent changes in calcium signalling may be pro- or anticonvulsant. In addition to neurons, CALHM1 has also been shown to be expressed in astrocytes [26], a cell type well-established to contribute to seizures [27]. Therefore, future research should carefully characterize in what brain region and what cell types CALHM1 gets activated during seizures and what down-stream signalling molecules (*e.g*., ATP) are involved.

Interestingly, CALHM1 protein levels seem to be downregulated in the hippocampus in epilepsy (mice and humans). Again, while we do not know the mechanisms, it is tempting to speculate this down-regulation to be a maladaptive pro-seizure response. It is, however, important to keep in mind that protein distribution and function can show marked differences between physiological conditions and diseases conditions (*i.e*., epilepsy), thus to proof a function of CALHM1 in epilepsy would require the treatment of mice post-IAKA once mice are epileptic.

While our study provides the proof-of-principle that CALHM1 is involved in the generation of seizures, there are several caveats that need to be considered. While we show a decrease in CALHM1 protein levels in the hippocampus of TLE patients, this could partly be attributed to post-mortem effects on protein expression. Experiments should be repeated in mice deficient in CALHM1 or using CALHM1-targeting siRNA [28], thereby excluding potential off-target effects of CGP37157. Moreover, future experiments should be designed to establish the mechanisms of action (*e.g*., cell-type specific expression and activation of CALHM1, CALHM1-dependent changes in intracellular Ca^2+^/eATP) and its role during different pathological contexts (*i.e*., acute seizures vs epilepsy).

In conclusion, our findings provide proof-of-principle that CALHM1 is involved in seizure generation. While our results highlight a previously unrecognized role for CALHM1 in modulating seizure severity, further studies in chronic epilepsy models are needed to define its precise function in epileptogenesis.

## Supplementary Information

Below is the link to the electronic supplementary material.

Supplementary file1 (DOCX 1094 KB)

Supplementary file2 (DOCX 28 KB)

## Data Availability

Data is provided within the manuscript or supplementary information files.
