# Supplementary material for: The CALHM1 blocker CGP37157 increases seizure severity during status epilepticus in adult mice
Source: Purinergic Signal. 2025 Jul 2;21(6):1241–8. doi: 10.1007/s11302-025-10103-9 (PMC12722193; doi:10.1007/s11302-025-10103-9)
Supplement: Supplementary file 1 — Supplementary file1 (DOCX 1094 KB) [file 11302_2025_10103_MOESM1_ESM.docx]

**Supplementary Material**

**
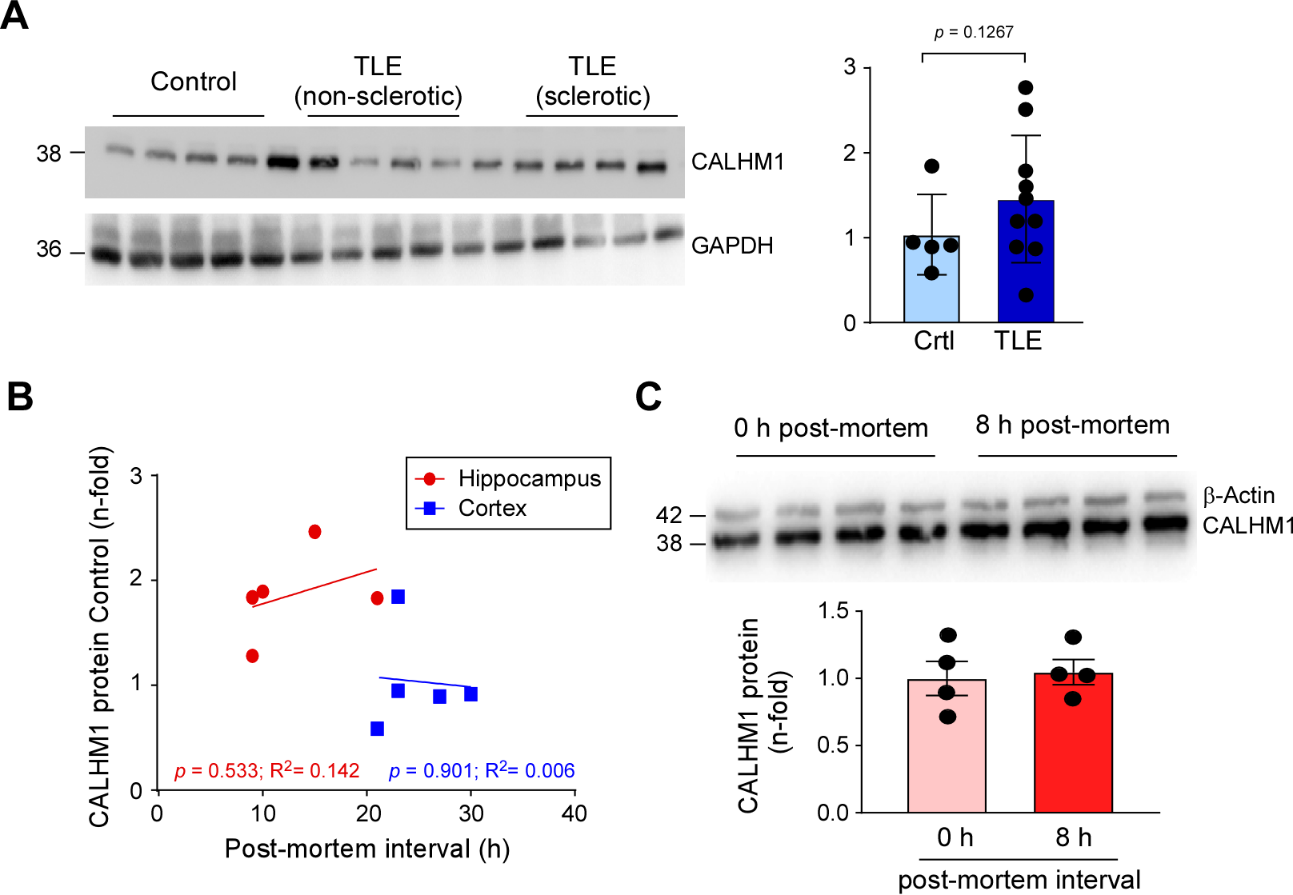
**

**Supplementary Figure 1: *CALHM1 protein levels in the cortex of TLE patients*.** (**A**) Western blot and graphs showing CALHM1 protein levels in the cortex of healthy control and resected tissue from TLE patients with and without hippocampal sclerosis (N = 5 (control) and 10 (cortex). Data are shown as mean ± SD. (**B**) Graph showing no correlation between CALHM1 protein levels in the hippocampus and cortex of controls and post-mortem interval (N = 5 per group). (**C**) Simulated post-mortem experiment in mice: Western blot and corresponding graph showing similar hippocampal CALHM1 protein levels 8 h post-mortem. Mouse brains were either immediately frozen after killing mice or extracted 8 h after killing mice which were left at room temperature (N = 4 per group). β-Actin was used as loading control.

**
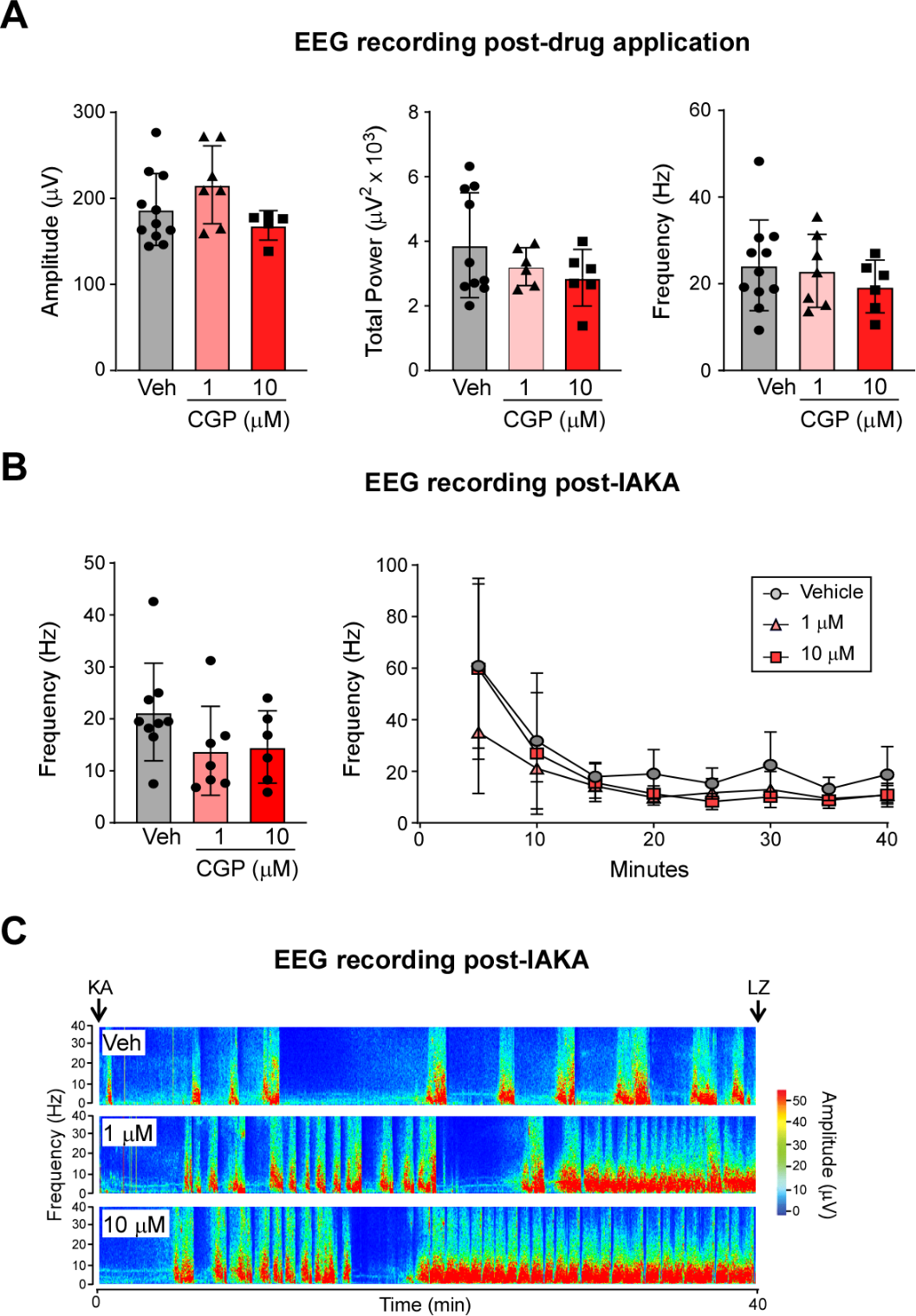
**

**Supplementary Figure 2: *EEG analysis following treatment with*** ***CGP37157*.** (**A**) Graphs showing amplitude, total power and frequency during a 5 in recording period between i.c.v. injection of CGP37157 and IAKA injections (N = 11 (Veh), 7 (1 µM) and 6 (10 µM). One value was identified as outlier (10 µM, amplitude) and excluded. (**B**) Graphs showing EEG frequency changes from the time of IAKA injection until treatment with lorazepam (left: EEG analysis of complete 40 min; right: EEG analysis of 5 min segments) (N = 9 (Veh), 7 (1 µM) and 6 (10 µM). (**C**) Representative EEG heat maps during IAKA-induced SE of the three treatment groups.
