# Supplementary material for: The CALHM1 blocker CGP37157 increases seizure severity during status epilepticus in adult mice
Source: Purinergic Signal. 2025 Jul 2;21(6):1241–8. doi: 10.1007/s11302-025-10103-9 (PMC12722193; doi:10.1007/s11302-025-10103-9)
Supplement: Supplementary file 2 — Supplementary file2 (DOCX 28 KB) [file 11302_2025_10103_MOESM2_ESM.docx]

|  |  |  |  |  |  |  |  |
| --- | --- | --- | --- | --- | --- | --- | --- |
|  | **Supplementary Table 1. Metadata for human cortex and hippocampus samples** | | | | | |  |
|  | **Identifier** | **Sex** | **Age (yrs)** | **Tissue** | **Primary cause of death** | **PMI (hours)** |  |
|  | C1 | F | 38 | Hippocampus | Undetermined | 9 |  |
|  | C2 | M | 54 | Hippocampus | Natural | 9 |  |
|  | C3 | F | 50 | Hippocampus | Complications of Lung Cancer | 15 |  |
|  | C4 | M | 59 | Hippocampus | multiple injuries | 10 |  |
|  | C5 | M | 43 | Hippocampus | Hypertensive Cardiovascular Disease | 21 |  |
|  | **Identifier** | **Sex** | **Age (yrs)** | **Tissue** | **Primary cause of death** | **PMI (hours)** |  |
|  | C1 | M | 43 | A21, Temporal cortex | Hypertensive Cardiovascular Disease | 21 |  |
|  | C2 | F | 34 | A21, Temporal cortex | Pulmonary Emboli with Deep Vein Thrombosis | 27 |  |
|  | C3 | F | 36 | A21, Temporal cortex | Natural | 23 |  |
|  | C4 | M | 39 | A21, Temporal cortex | Natural | 30 |  |
|  | C5 | F | 29 | A21, Temporal cortex | Accidental (Heroin Intoxication) | 23 |  |
|  | **Identifier** | **Sex** | **Age (yrs)** | **Tissue** | **Neuropathology** | **PMI (hours)** |  |
|  | TLE1 | F | 44 | Hippocampus | Non-Sclerotic | NA |  |
|  | TLE2 | M | 37 | Hippocampus | Non-Sclerotic | NA |  |
|  | TLE3 | M | 42 | Hippocampus | Non-Sclerotic | NA |  |
|  | TLE4 | F | 42 | Hippocampus | Non-Sclerotic | NA |  |
|  | TLE5 | M | 36 | Hippocampus | Non-Sclerotic | NA |  |
|  | TLE6 | F | 47 | Hippocampus | Hippocampal sclerosis | NA |  |
|  | TLE7 | F | 46 | Hippocampus | Hippocampal sclerosis | NA |  |
|  | TLE8 | M | 47 | Hippocampus | Hippocampal sclerosis | NA |  |
|  | TLE9 | F | 48 | Hippocampus | Hippocampal sclerosis | NA |  |
|  | TLE10 | M | 49 | Hippocampus | Hippocampal sclerosis severe/normal temporal neocortex | NA |  |
|  | **Identifier** | **Sex** | **Age (yrs)** | **Tissue** | **Neuropathology** | **PMI (hours)** |  |
|  | TLE1 | F | 44 | Cortex | Cortical dysplasia | NA |  |
|  | TLE2 | M | 37 | Cortex | Not provided | NA |  |
|  | TLE3 | M | 42 | Cortex | No evidence of cortical dysplasia, neoplasm, inflammation or vascular malformation. | NA |  |
|  | TLE4 | F | 42 | Cortex | Not provided | NA |  |
|  | TLE5 | M | 36 | Cortex | Not provided | NA |  |
|  | TLE6 | F | 47 | Cortex | Not provided | NA |  |
|  | TLE7 | F | 46 | Cortex | Not provided | NA |  |
|  | TLE8 | M | 47 | Cortex | Not provided | NA |  |
|  | TLE9 | F | 48 | Cortex | Not provided | NA |  |
|  | N/A: Non-applicable; PMI: Post-Mortem Interval | | | | | |  |
|  |  |  |  |  |  |  |  |
